# Sepsis-Induced Cardiomyopathy Secondary to Escherichia coli Sepsis and Intestinal Perforation: A Diagnostic Challenge in a Paediatric Patient

**DOI:** 10.7759/cureus.76552

**Published:** 2024-12-29

**Authors:** Himanshu Kumar, Shishir Kumar, Deb Sanjay Nag, Kumar Diwakar, Niharika Singh

**Affiliations:** 1 Department of Anaesthesiology, Tata Main Hospital, Jamshedpur, IND; 2 Department of Surgery, Tata Main Hospital, Jamshedpur, IND; 3 Department of Anesthesiology, Tata Main Hospital, Jamshedpur, IND; 4 Department of Paediatrics, Tata Main Hospital, Jamshedpur, IND

**Keywords:** abdominal sepsis, intestinal perforation, septic cardiomyopathy, septic shock in children, urgent laparotomy

## Abstract

A five-year-old male presented with abdominal pain, fever, vomiting, and constipation. Initial investigations suggested subacute intestinal obstruction. Laparotomy revealed intestinal perforation with peritonitis due to *Escherichia coli*. Post-operatively, the patient developed sepsis-induced cardiomyopathy (SICM), requiring inotropic support and mechanical ventilation. The complex clinical presentation and overlap of symptoms with septic shock delayed the diagnosis of SICM, making timely identification challenging. This case highlights the diagnostic challenges in identifying sepsis-induced cardiomyopathy in the context of severe sepsis and abdominal pathology, underscoring the need for early recognition, particularly using echocardiography for myocardial dysfunction assessment.

## Introduction

*Escherichia coli* is a frequent cause of gastrointestinal infections, sometimes leading to severe sepsis and potentially life-threatening complications [1]. Despite the paucity of literature on sepsis-induced cardiomyopathy (SICM), it is understood to be a complication of severe sepsis characterized by reversible myocardial dysfunction [2]. Sepsis-induced myocardial dysfunction (SMD) was associated with significantly higher mortality (54.5% vs. 7.5%, p<0.001), increased organ dysfunction, and worse overall outcomes in children with septic shock [3]. The underlying pathophysiology includes the release of inflammatory cytokines, altered ion channel function, and myocardial dysfunction, which is critical in understanding its development in the context of *E. coli *sepsis. It was also an independent predictor of death [3]. Early recognition and aggressive management are crucial for optimal outcomes. Sepsis-induced cardiomyopathy (SICM) in paediatric patients presents unique diagnostic challenges. In paediatric patients, SICM presents differently due to unique developmental factors and often requires a more nuanced approach to diagnosis and management compared to adults. This report describes a case of SICM secondary to intestinal perforation and *E. coli* sepsis in a pediatric patient, highlighting the diagnostic challenges encountered and situational awareness needed to manage such patients. Though limited, the available literature on SICM in paediatric populations reveals important clinical trends, especially concerning its association with septic shock and subsequent cardiac dysfunction. 

## Case presentation

A five-year-old male child, weighing 19 kilograms, presented with a 10-day history of abdominal pain, a one-week history of fever, three to four episodes per day of vomiting for three days, and two days of constipation. The patient was febrile (temperature 100.2°F), tachycardic with a heart rate of 156 beats per minute (bpm), and hypotensive (blood pressure 92/56 mmHg). Respiratory rate was 36 breaths/minute, with oxygen saturation (SpO_2_) 98% on room air. Physical examination revealed abdominal distension, guarding, rigidity, and tenderness all over the abdomen. The clinical examination was suggestive of gross peritonitis.

Laboratory investigations revealed a haemoglobin of 11.1 grams per decilitre (g/dL) and a raised total leucocyte count (TLC) of 14470 white blood cells per microliter of blood (with a neutrophilic preponderance of 81%). Serum electrolytes were within the normal range. C-reactive protein (CRP) was elevated at 18.27 milligrams per decilitre (mg/dL) (Table 1, Table 2).

**Table 1:** Complete blood count.

Complete blood count		
Investigation	Patient’s lab values	Reference range
Hemoglobin	11.1 g/dL	11.5-16.5 g/dL
White blood cell count	14.4×10^3 ^/µL	4×10^3 ^-11×10^3 ^/µL
Hematocrit	47%	35.0-50.0%
Mean corpuscular volume	84.1 fL	70-90 fL
Mean corpuscular hemoglobin concentration	29.3 g/dL	31.5-34.5 g/dL
Platelet count	2.66×10^3 ^/µL	1.5-4.5×10^3 ^/µL

**Table 2:** Metabolic parameters.

Metabolic parameters		
Investigation	Patient’s lab values	Reference range
Serum creatinine	0.39 mg/dL	0.3-0.7 mg/dL
Serum sodium	132 mEq/L	138-145 mEq/L
Serum potassium	3.9 mEq/L	3.5-5.5 mEq/L
Total bilirubin	0.56 mg/dL	0.2-1.0 mg/dL
Conjugated bilirubin	0/17 mg/dL	0.1-0.5 mg/dL
Unconjugated bilirubin	0.39 mg/dL	0.1-0.7 mg/dL
Alanine transaminase	20.8 U/L	5-45 U/L
Aspartate transaminase	32.8 U/L	0-35 U/L
Alkaline phosphatase	115.8 U/L	53-141 U/L
Amylase	68.5 U/L	60-100 U/L
Lipase	13.0 U/L	11-36 U/L
Prothrombin time (Test)	17. 9 seconds	10.8-14.6 seconds
Mean normal prothrombin time	-	11.7-15.4 seconds
International normalized ratio	1.44	0.8-1.2

**Figure 1:**
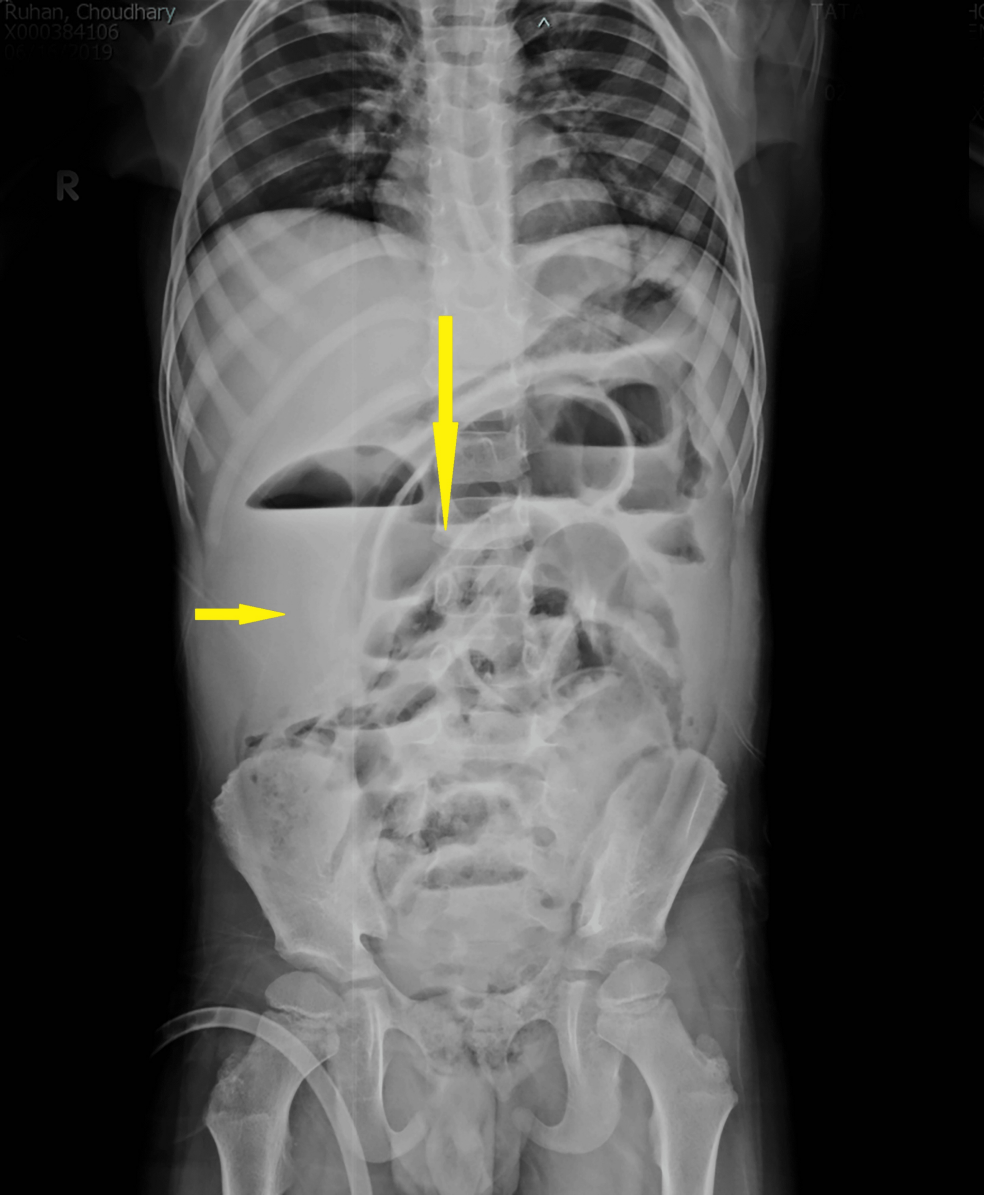
Erect X-ray of the abdomen. Collection in the right iliac fossa (horizontal arrow) and periumbilical region, along with clumped-up small bowel loops (vertical arrow).

An erect X-ray of the abdomen revealed a collection in the right iliac fossa and periumbilical region, along with clumped-up small bowel loops. Figure 1 shows collection in the right iliac fossa and periumbilical region, along with a clumped-up small bowel.

**Figure 2:**
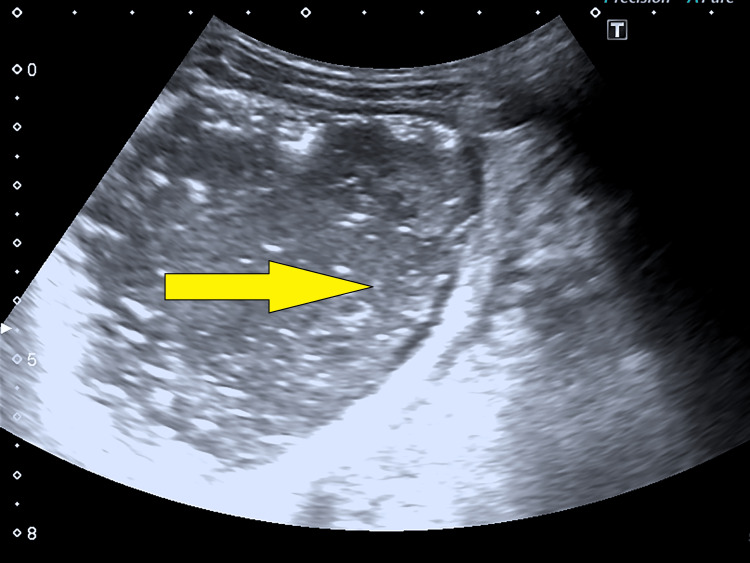
Abdominal ultrasonography showing free fluid in the abdomen with septations, suggestive of pyoperitoneum.

A posteroanterior (PA) chest X-ray did not reveal any abnormal findings. A bedside ultrasound of the abdomen suggested free fluid in the abdomen with septations, suggestive of pyoperitoneum. Figure 2 shows free fluid in the abdomen with septations, suggestive of pyoperitoneum.

The patient was administered intravenous cefoperazone+sulbactam, amikacin, and metronidazole to cover for all suspected organisms before the availability of a definitive culture and sensitivity reports, along with an intravenous crystalloid. An emergency laparotomy under general anesthesia was planned considering the above clinical and radiological findings. During the surgery under general anesthesia with nitrous oxide/oxygen/sevoflurane as inhalational agents, intravenous fentanyl for analgesia, and vecuronium for neuromuscular blockade, we found dense adhesions between the bowels, an interloop abscess, a pelvic abscess, and a 1.5 cm perforation in the transverse colon. The right subhepatic area contained approximately 200 ml of pus, and the pelvic cavity contained faecal pellets. Additionally, there was inflammation in the appendix and the presence of omental caking. Primary closure of the transverse colon perforation, resection of the 5 cm pregangrenous ileum, a double-barrel ileostomy, and appendicectomy were done. An extrahepatic drain was placed to manage the pus collection. We sent the pus for culture, sensitivity, and the cartridge-based nucleic acid amplification test (CBNAAT) to detect *Mycobacterium tuberculosis*. We sent the omentum, appendix, and resected ileum for histopathological examination.

**Figure 3:**
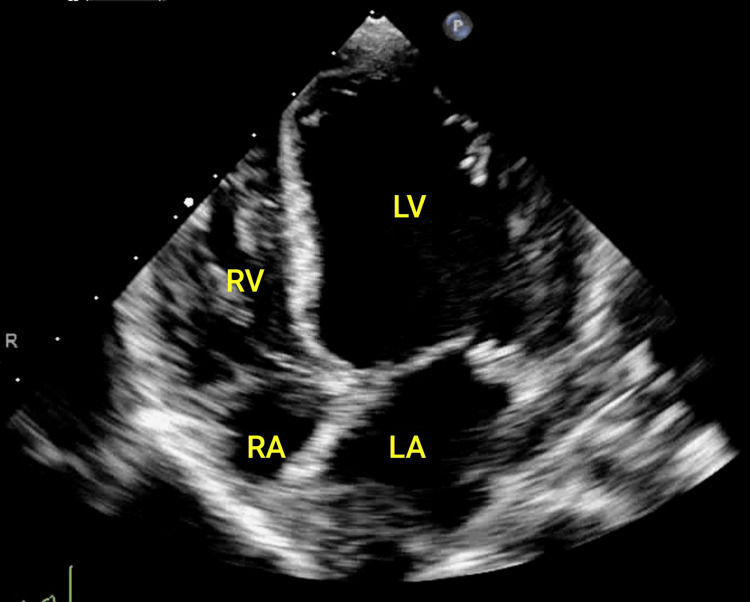
Transthoracic echocardiographic apical four chamber view, showing dilated left ventricle and atrium. LV: Left ventricle, LA: Left atrium, RV: Right ventricle, RA: Right atrium.

Intraoperative blood loss was approximately 100 ml. The patient continued to experience hypotension and sinus tachycardia (heart rate: 158-190 beats per minute) intraoperatively with low urine output, despite adequate fluid resuscitation. Following surgery, the patient continued to experience hemodynamic instability, requiring inotropic support and mechanical ventilation. In the paediatric intensive care unit (PICU), the patient received multiple fluid boluses (40 ml/kg), inotropic support with norepinephrine (0.2 mcg/kg/min), and epinephrine (0.1 mcg/kg/min). Despite the inotropic support, shock persisted. Intravenous hydrocortisone (38 mg) was initiated due to fluid refractory and vasopressor-dependent shock. Although initially attributed to septic shock, the patient’s persistent hypotension and tachycardia, despite adequate fluid resuscitation, prompted further investigation for potential myocardial involvement. Despite the initial focus on managing sepsis, the development of cardiac dysfunction became apparent through echocardiographic findings. A 2D echocardiogram revealed a dilated left atrium and left ventricle, along with a reduced ejection fraction of 40%, indicative of sepsis-induced cardiomyopathy. Figure 3 shows the dilated left atrium and left ventricle.

There was no vegetation or pericardial effusion. We added an intravenous infusion of milrinone (0.5 mcg/kg/min). The echocardiogram findings and poor response to inotropic support led to the diagnosis of sepsis-induced cardiomyopathy (SICM). Blood and urine cultures were negative, while pus cultures detected *E. coli* sensitive to both cefoperazone+sulbactam and amikacin. Intravenous antibiotics were continued. Histopathological examination of the specimens received from the omentum, appendix, and ileum revealed nonspecific acute inflammatory pathology and no evidence of granulomatous inflammation, fungal hyphae, or malignancy.

We titrated inotropic support to maintain an acceptable mean arterial blood pressure for the age. A repeat echocardiogram two days later showed an improved left ventricular ejection fraction of 58%. Inotropes and milrinone were gradually tapered over the next three days. We started Ryle's tube feed on the third postoperative day and removed the extrahepatic drain on the fifth postoperative day. The patient was gradually weaned off mechanical ventilation, and tracheal extubation was done on the seventh postoperative day. No further end-organ support was required. The patient was discharged from the hospital on the 10th postoperative day without any specific complaints or symptoms. The patient was regularly followed up in the surgical OPD and subsequently underwent an uneventful elective ileostomy closure six weeks later. The patient was followed subsequently for the next six months after the elective colostomy closure without any clinical complaints.

## Discussion

The patient initially presented with abdominal pain, vomiting, and fever. Over the following days, his condition worsened, and upon surgical intervention, a perforated colon and peritonitis were diagnosed. Following surgery, persistent hypotension and tachycardia led to the suspicion of SICM, which was confirmed by echocardiography. 

Sepsis-induced cardiomyopathy (SICM) is a well-recognized complication of severe sepsis and septic shock, particularly in children, where it can present with significant diagnostic challenges due to overlapping symptoms with other conditions. SICM is a recognized complication of sepsis, thought to be mediated by the release of inflammatory cytokines. First described by Parker MM et al. in 1984, while the cardiac function of the survivors improved over the next seven-ten days, the non-survivors did not report any improvement in cardiac function (ejection fraction) over the period [4]. Although the incidence of left ventricular systolic dysfunction in septic shock varies between 23% and 65% in adults, the incidence in pediatric patients is still underresearched. This case illustrates the importance of recognizing SICM in children, as early identification can significantly affect treatment outcomes. In pediatric patients, SICM is often underdiagnosed or misdiagnosed due to the absence of clear, standardized criteria [4].

SICM is characterized by left ventricular dilation with normal or low filling pressures and increased compliance, leading to excessive volume increases with fluid resuscitation [4]; reduced left (and likely right) ventricular ejection fraction [5-7]; generally reversible dysfunction, with improvement typically within seven to ten days [4,8].

Currently, there's no universally accepted definition or diagnostic criteria for SICM. Studies typically diagnose SICM by excluding pre-existing heart conditions and using echocardiographic indicators, primarily left ventricular ejection fraction (LVEF) [9]. However, the LVEF threshold varies considerably across studies, ranging from<40% to <50%. Some studies also incorporate cardiac index as a diagnostic criterion [9]. However, the dearth of literature on paediatric SICM is even greater.

The mechanisms behind SICM involve a combination of systemic inflammatory responses and myocardial changes [10]. The release of inflammatory cytokines and myocardial depressant factors leads to decreased cardiac function. These factors, in turn, cause the reduction in ejection fraction and left ventricular dilation observed in SICM. Elevated cardiac troponin I levels correlate with reduced cardiac function and disease severity [10]. In children, who often present with a higher inflammatory response to sepsis, distinguishing between septic shock and SICM can be challenging. Critical care echocardiography is the definitive diagnostic tool for sepsis-induced cardiomyopathy (SCM) and should be used to assess all hemodynamically unstable patients [11]. In children, echocardiography remains the gold standard for diagnosing SICM, but interpreting findings like a reduced ejection fraction can be challenging due to the differences in normal pediatric cardiac physiology.

As for septic shock, initial treatment is dependent on early suspicion and diagnosis, followed by fluid expansion and administration of vasopressors [11]. Early studies primarily in pediatric populations suggested potential cardiovascular benefits with milrinone and amrinone in septic shock [12]. Although limited data exist on the use of milrinone in adult sepsis, early studies in pediatric populations have suggested it improves cardiac output and tissue perfusion. Although considered an effective treatment modality in select “pediatric patients with septic shock” [13] as far back as 1996, its use has not been very common despite various isolated studies that have shown its effectiveness in improving cardiac output and tissue perfusion [14]. In this case, milrinone played a pivotal role in stabilizing the patient’s hemodynamics and improving cardiac function. 

Inotropic support should be considered for sepsis and septic shock patients exhibiting myocardial dysfunction or persistent hypoperfusion, even after adequate fluid resuscitation and vasopressor therapy to achieve target mean arterial pressure [12]. This case demonstrates that early identification of SICM, particularly in children with severe sepsis and abdominal pathology, is critical for improving outcomes. Clinicians should maintain a high level of suspicion for myocardial dysfunction in patients with septic shock who do not respond to fluid resuscitation alone. 

## Conclusions

SICM is a sepsis complication characterized by left ventricular dysfunction, typically reversible within seven to ten days. The etiology is complex, involving inflammatory cytokines, altered ion channel function, and myocardial structural changes. Early identification of SICM in children, particularly those with sepsis and septic shock, is crucial, as timely treatment can significantly improve outcomes. The lack of standardized diagnostic criteria for SICM, particularly in children, poses significant challenges. Early echocardiography and a high index of suspicion are critical for diagnosis and management. The diagnosis relies on echocardiography and excludes pre-existing heart conditions.

Inotropic support, including medications like milrinone, should be considered in pediatric septic shock with myocardial dysfunction, especially when conventional treatments fail. This case underscores the importance of early recognition of SICM in pediatric sepsis. Clinicians should maintain a high level of suspicion for cardiac involvement in children presenting with septic shock who exhibit poor response to fluid resuscitation. Given the limited data on pediatric SICM, further research is needed to establish clearer diagnostic criteria and optimal treatment strategies for this population. 
